# Genetic Variability of Antioxidative Mechanisms and Cardiotoxicity after Adjuvant Radiotherapy in HER2-Positive Breast Cancer Patients

**DOI:** 10.1155/2020/6645588

**Published:** 2020-12-19

**Authors:** Tanja Marinko, Jakob Timotej Stojanov Konda, Vita Dolžan, Katja Goričar

**Affiliations:** ^1^Institute of Oncology Ljubljana, Ljubljana, Slovenia; ^2^University of Ljubljana, Faculty of Medicine, Ljubljana, Slovenia; ^3^University of Ljubljana, Faculty of Medicine, Institute of Biochemistry and Molecular Genetics, Pharmacogenetics Laboratory, Ljubljana, Slovenia

## Abstract

**Background:**

Breast cancer treatment is associated with the occurrence of various cardiac adverse events. One of the mechanisms associated with cardiotoxicity is oxidative stress, against which cells are protected by antioxidative enzymes. Genetic variability of antioxidative enzymes can affect enzyme activity or expression, which modifies the ability of cells to defend themselves against oxidative stress and could consequently contribute to the occurrence of treatment-related cardiotoxicity. Our aim was to evaluate the association of common polymorphisms in antioxidative genes with cardiotoxicity after adjuvant radiotherapy (RT) in HER2-positive breast cancer patients.

**Methods:**

Our retrospective study included 101 HER2-positive early breast cancer patients who received trastuzumab and adjuvant RT. We isolated DNA from buccal swabs and used competitive allele-specific PCR for genotyping of *PON1* rs854560 and rs662, *GSTP1* rs1138272 and rs1695, *SOD2* rs4880, *CAT* rs1001179, and *HIF1* rs1154965 polymorphisms. N-terminal pro B-type natriuretic peptide (NT-proBNP), left ventricular ejection fraction, and NYHA class were used as markers of cardiotoxicity. We used logistic regression to evaluate the association of genetic factors with markers of cardiotoxicity.

**Results:**

Carriers of at least one polymorphic *PON1* rs854560 allele were less likely to have increased NT-proBNP (OR = 0.34; 95% CI = 0.15-0.79; *P* = 0.012), even after adjustment for age (OR = 0.35; 95% CI = 0.15-0.83; *P* = 0.017). Carriers of at least one polymorphic *PON1* rs662 allele were more likely to have increased NT-proBNP (OR = 4.44; 95% CI = 1.85-10.66; *P* = 0.001), even after adjustment for age (OR = 5.41; 95% CI = 2.12-13.78; *P* < 0.001). *GSTP1* rs1695 was also associated with decreased NT-proBNP in the multivariable analysis (*P* = 0.026), while *CAT* rs1001179 was associated with NYHA class in the univariable (*P* = 0.012) and multivariable analysis (*P* = 0.023).

**Conclusion:**

In our study, polymorphisms *PON1* rs662 and rs854560, *CAT* rs1001179, and *GSTP1* rs1695 were significantly associated with the occurrence of cardiac adverse events after adjuvant RT and could serve as biomarkers contributing to treatment personalization.

## 1. Introduction

Adjuvant radiotherapy (RT) has significantly improved disease-specific survival for patients with early-stage breast cancer [1, 2]. As a consequence, more cancer survivors may experience late complications of treatment [3]. Radiation dose received by the heart during adjuvant RT of breast or thoracic wall may result in a range of cardiotoxic effects including coronary artery disease, cardiomyopathy, pericardial disease, valvular dysfunction, and conduction abnormalities [4, 5]. There is no minimum radiation dose to the heart that is entirely safe [4].

A combination of adjuvant RT and systemic oncological treatment may have an even worse impact on the cardiac-related outcome [6]. This combination is frequently used in HER2-positive breast cancer, a subtype of breast cancer with amplification or overexpression of the human epidermal growth factor receptor 2 (HER2) oncogene, which represents approximately 15% of all breast cancers [7, 8]. In standard clinical practice, this subtype of breast cancer is treated with a least two types of cardiotoxic systemic treatment [7, 9]. Anthracyclines are prescribed as a part of neoadjuvant or adjuvant chemotherapy and are followed by anti-HER2 treatment with trastuzumab, a monoclonal antibody [9]. Both types of treatment increase the survival of HER2-positive breast cancer patients but are cardiotoxic [10–13]. Anthracycline-related cardiotoxicity results, at least to some degree, in myocyte destruction and clinical heart failure and is irreversible. Trastuzumab-related cardiotoxicity is most often manifested by an asymptomatic decrease in left ventricular ejection fraction (LVEF) and less often by clinical heart failure [13–15].

Different biomarkers and imaging techniques and their potential role in monitoring cardiotoxicity have already been evaluated [6]. The use of blood-based biomarkers to detect radiation or systemic treatment-induced cardiotoxicity is very promising as it is minimally invasive, affordable, and repeatable [16]. Currently, the determination of LVEF and N-terminal pro-brain natriuretic peptide (NT-proBNP) is mostly used for monitoring cardiotoxicity of cancer treatment [14, 17, 18]. LVEF is the golden standard for monitoring cardiac function in patients receiving cardiotoxic therapy. Echocardiography and radionuclide ventriculography are imaging techniques that are being most widely used in this setting for the assessment of LVEF [14].

Brain natriuretic peptide (BNP), a member of the family of natriuretic hormones, seems to be one of the most appropriate biomarkers for cardiotoxicity evaluation [19, 20]. After being synthesized, its inactive form is then cleaved into active BNP and inactive NT-proBNP. NT-proBNP is a sensitive biomarker of both systolic and diastolic heart failure [21]. NT-proBNP was also an early and sensitive diagnostic and prognostic biomarker for the evaluation of cardiotoxicity of cancer chemotherapy and RT [20, 22, 23]. Patients with elevated NT-proBNP had a higher possibility of asymptomatic LVEF reduction or developing symptomatic heart failure later on. Because changes in NT-proBNP usually occur earlier than changes in LVEF, its elevated level exposes patients at higher risk.

One of the molecular mechanisms associated with cancer treatment response and occurrence of adverse events is oxidative stress. Oxidative stress occurs as a result of excess formation of reactive oxygen species (ROS) that can cause a variety of cellular damage, including DNA modifications, breaks, deletions and translocations, lipid peroxidation, amino acid modifications, and protein conformational changes [24]. Oxidative stress is also among the key stimuli leading to carcinogenesis [25]. RT can trigger oxidative stress in cancer cells, causing DNA damage and stress response activation in mitochondria and the endoplasmic reticulum. Increased ROS formation affects both cancer and surrounding healthy cells, leading to various adverse events, including cardiotoxicity [26, 27]. Oxidative stress was also proposed as one of the mechanisms involved in anthracycline and trastuzumab cardiotoxicity [28–30]. Anthracyclines increase ROS formation through various enzymatic and nonenzymatic reactions [28, 30]. For example, the reduction of anthracyclines results in the formation of semiquinone free radicals and increased formation of ROS through different enzymes. Anthracyclines also interact with ferric iron, leading to altered redox-cycling and the formation of superoxide anion [30]. Trastuzumab is associated with increased ROS formation, decreased glutathione concentration, and decreased activity of antioxidative enzymes in cell lines [31]. Studies suggest trastuzumab mostly affects oxidative stress due to dysregulated HER2 signalling through mitogen-activated protein kinase, phosphoinositide 3-kinase, and neuregulin signalling pathways leading to increased ROS formation [29, 30].

Antioxidative enzymes are part of the cellular mechanisms maintaining appropriate levels of ROS and could therefore affect treatment-related cardiotoxicity. Among them are glutathione-S-transferases (GSTs), crucial for detoxification of endogenous and exogenous substrates by conjugation with reduced glutathione. The most important subtypes are GSTM1, GSTT1, and GSTP1 [32]. The enzyme superoxide dismutase (SOD) catalyzes the conversion of superoxide radical to hydrogen peroxide [33], after which the latter can be converted to water, catalyzed by the enzyme catalase [34]. Another antioxidative enzyme is paraoxonase 1 (PON1) that has organophosphates, lactonase, and esterase activity and is located in high-density lipoproteins (HDL) [35]. Oxidative stress was also proposed as a modulator of hypoxia-inducible factor 1 (HIF1) activity [36, 37], a transcription factor involved in response to hypoxia that regulates the expression of several genes involved in important cell processes and diseases [38, 39].

There are significant differences in the occurrence of postirradiation toxicity among breast cancer patients, and genetic factors may contribute to the observed interindividual variability [40–42]. Several genetic polymorphisms affect the activity or expression of antioxidative enzymes [32, 39, 43–45] and could consequently also affect the occurrence of treatment-related cardiotoxicity.

The aim of our study was therefore to evaluate whether common polymorphisms in antioxidative genes affect the cardiotoxicity after adjuvant RT in HER2-positive early breast cancer patients.

## 2. Patients and Methods

### 2.1. Patients

Our retrospective study included patients with human epidermal growth factor receptor-2- (HER2-) positive left- or right-sided early breast cancer (stage I-III), treated concurrently with trastuzumab and RT at the Institute of Oncology Ljubljana between June 2005 and December 2010. HER2 status of the tumour was determined according to our standard clinical practice [8]. All patients were treated according to the clinical guidelines with surgery, chemotherapy, endocrine therapy in case of hormone receptor-positive disease, trastuzumab, and RT. Trastuzumab treatment started before RT or on the first day of RT at the latest. After the adjuvant treatment with RT and trastuzumab, a follow-up clinical examination was performed. All patients also filled out questionnaires about smoking, concomitant diseases, and problems related to cardiovascular diseases. All other data were obtained from the patients' records.

The study was approved by the Republic of Slovenia National Medical Ethics Committee (approval number 39/05/15, 0120-54/2015-2) and was internationally registered at ClinicalTrial.gov (identifier NCT01572883). The study was conducted in accordance with the Declaration of Helsinki. All participants signed an informed consent before participating in the study.

#### 2.1.1. Systemic Treatment

Data regarding systemic therapy were obtained from the patient's individual medical record. Most patients were treated with one of the chemotherapy regimens that include anthracyclines and taxanes and were used in standard clinical practice at the time of the treatment. Mostly used treatment schemes were as follows: Option 1: 4 cycles of epirubicin plus cyclophosphamide (EC) or doxorubicin plus cyclophosphamide (AC) every 3 weeks, followed by 12 cycles of paclitaxel weekly; Option 2: 4 cycles of EC or AC every 3 weeks, followed by 3 cycles of docetaxel every 3 weeks; or Option 3: 3 to 4 cycles of 5-fluorouracil, epirubicin, and cyclophosphamide (FEC) or doxorubicin in combination with 5-fluorouracil and cyclophosphamide (FAC) every 3 weeks, followed by 3-4 cycles of docetaxel every 3 weeks. The criteria for adjuvant treatment with trastuzumab regarding tumour, nodal stage, and cardiac function were the same as in pivotal adjuvant trials: tumours larger than 2 cm if node-negative disease, any tumour size if node-positive disease, WHO performance status zero or one, no serious concomitant cardiac disease, and treatment with adjuvant chemotherapy [46]. Treatment with trastuzumab started 3 weeks after the last cycle of anthracyclines and was prescribed for 1 year.

#### 2.1.2. Locoregional Treatment

According to clinical guidelines patients were operated with either breast conservation surgery or mastectomy and either sentinel node biopsy or axillary dissection. After the operation and chemotherapy, they were irradiated using two-dimensional (2D RT) or three-dimensional conformal RT (3D CRT). Some of the patients received electron-beam chest wall irradiation. Whole breast RT was required in all patients who underwent breast cancer surgery. In addition to the irradiation of the breast/chest wall, all patients with 4 or more positive axillary lymph nodes also received regional RT.

Patients were irradiated with a total dose (TD) = 25 × 2 Gy, 5 fractions per week. A minority received RT with TD = 17 or 18 × 2.5 Gy, 5 fractions per week. RT was performed 3 or more weeks after chemotherapy had been completed and concurrently with trastuzumab treatment as well as hormonal therapy in case of hormone receptor-positive breast cancer.

### 2.2. Assessment of Cardiotoxicity

Patients were classified according to New York Heart Association (NYHA) classification to assess signs of heart failure [47].

#### 2.2.1. Echocardiography and Radionuclide Ventriculography

Echocardiography with LVEF measurement was performed before adjuvant RT and after the completed treatment with RT and trastuzumab. Baseline LVEF was determined either by using echocardiography or radionuclide ventriculography as previously described [8]. Normal range for LVEF was 50% or more. The difference between both LVEF measurements was analysed. Absolute change in LVEF was calculated as the difference between LVEF after completed adjuvant RT and trastuzumab treatment, and LVEF before RT. Important LVEF reduction was classified as a decrease of LVEF for ≥10% or a final value of LVEF <50% [17].

#### 2.2.2. NT-proBNP

NT-proBNP was determined with the Cobas e 411 analyser (Roche) according to our standard clinical practice at the follow-up clinical examination after the adjuvant treatment with RT and trastuzumab [8]. According to the instructions of the manufacturer, the values of NT-proBNP below 125 ng/l exclude heart dysfunction [48].

### 2.3. DNA Extraction and Genotyping

Genomic DNA was extracted from buccal swabs (INFINITI Buccal Sample Collection Kit, AutoGenomics Inc., Vista, CA, USA) using QIAamp DNA Mini Kit (QIAGEN, Hilden, Germany) according to the manufacturer's instructions. Common putatively functional single nucleotide polymorphisms (SNPs) in antioxidative genes *GSTP1*, *CAT*, *SOD2*, *PON1*, and *HIF1* were selected based on literature search. Genotyping was performed using fluorescent-based competitive allele-specific polymerase chain reaction (KASP, LGC Genomics, UK) following the manufacturer's instructions.

### 2.4. Statistical Analysis

Continuous and categorical variables were described using median with interquartile range (25%–75%) and frequencies, respectively. A dominant genetic model was used in the analyses. Deviation from Hardy-Weinberg equilibrium (HWE) was evaluated using chi-square test, and SNPs not in HWE were excluded from further analyses. To evaluate the association of selected SNPs with markers of cardiotoxicity, univariable and multivariable logistic regression were used to calculate odds ratios (ORs) and 95% confidence intervals (CIs). Clinical parameters used for adjustment were selected using stepwise forward-conditional logistic regression. Fisher's exact test was used to compare genotype frequencies if there were no patients in one of the categories. The nonparametric Mann-Whitney test was used to compare the distribution of continuous variables. The statistical analyses were carried out by using IBM SPSS Statistics version 21.0 (IBM Corporation, Armonk, NY, USA). To determine the combined effect of more SNPs within one gene, we reconstructed haplotypes using Thesias with the most common haplotype serving as a reference [49]. All statistical tests were two-sided, and the level of significance was set at 0.05.

## 3. Results

We included 101 HER2-positive early breast cancer patients treated with adjuvant RT and trastuzumab. Regarding systemic treatment, 99 (98.0%) patients were also treated with anthracyclines. Additionally, 58 (57.4%) received taxanes and 57 (56.4%) received hormonal therapy. Median time between the first and the last anthracycline application was 67 (51-105) days. Median time between the first and the last taxane application was 43 (42-75) days. Most patients (84, 83.2%) were irradiated with a total dose of 25 × 2 Gy, 5 fractions per week. All treatment was administered according to clinical guidelines. A total of 48 (47.5%) patients were operated and received RT on the left side. Detailed patients' characteristics and treatment parameters are presented in Table 1.

Median follow-up after diagnosis was 4.5 (3.2-5.9) years, and median follow-up after the onset of RT was 4.0 (2.6-5.4) years. Cardiotoxicity was evaluated in all patients after the treatment (Table 2). Median NT-proBNP level was 90 (56-157) ng/l. In total, 36 (35.6%) patients had increased NT-proBNP values with above 125 ng/l. Most patients did not exhibit signs of heart failure according to NYHA classification. After treatment, 17 (16.8%) patients had mild symptoms (NYHA class 2). LVEF measurements before and after RT were similar with the median change of 3 (-3 to 9) %. Important LVEF reduction was observed in 9 (8.9%) patients (Table 2).

All patients were genotyped for *PON1* rs854560 (p.Leu55Met), *PON1* rs662 (p.Gln192Arg), *GSTP1* rs1138272 (p.Ala114Val), *GSTP1* rs1695 (p.Ile105Val), *SOD2* rs4880 (p.Ala16Val), *CAT* rs1001179 (c.-330C>T), and *HIF1A* rs1154965 (p.Pro582Ser). Genotype and minor allele frequencies are presented in Supplementary Table 1. As *SOD2* rs4880 genotype distribution was not in agreement with HWE (*P* = 0.004), this SNP was excluded from further analyses. Genotype distributions of all other SNPs were in agreement with HWE.

### 3.1. NT-proBNP

**Figure 1:**
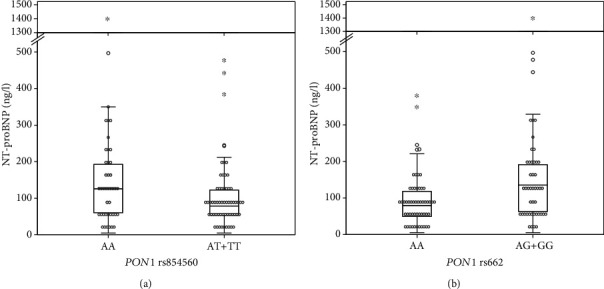
The association of *PON1* rs854560 (a) and *PON1* rs662 (b) polymorphisms with N-terminal pro B-type natriuretic peptide (NT-proBNP) levels after adjuvant radiotherapy in HER2-positive breast cancer patients.

Carriers of at least one polymorphic *PON1* rs854560 allele had significantly lower median NT-proBNP level (*P* = 0.048, Table 3, Figure 1(a)), while carriers of at least one polymorphic *PON1* rs662 allele had significantly higher median NT-proBNP level (*P* = 0.007, Table 3, Figure 1(b)).

Among clinical parameters, only higher age was significantly associated with increased NT-proBNP in our study group (OR = 0.61, 95% CI = 0.27-1.38, *P* = 0.231). Surgical treatment and RT side was not associated with increased NT-proBNP (right vs. left: OR = 1.05, 95% CI = 1.01-1.09, *P* = 0.023). None of the other treatment parameters, including chemotherapy parameters, or comorbidities were significantly associated with NT-proBNP (all *P* > 0.05).

Carriers of at least one polymorphic *PON1* rs854560 allele were significantly less likely to have increased NT-proBNP (OR = 0.34, 95% CI = 0.15-0.79, *P* = 0.012), even after adjustment for age (OR = 0.35, 95% CI = 0.15-0.83, *P* = 0.017) (Table 3). Carriers of at least one polymorphic *PON1* rs662 allele were significantly more likely to have increased NT-proBNP (OR = 4.44, 95% CI = 1.85-10.66, *P* = 0.001). This association remained significant even after adjustment for age (OR = 5.41, 95% CI = 2.12-13.78, *P* < 0.001). Additionally, carriers of at least one polymorphic *GSTP1* rs1695 were less likely to have increased NT-proBNP, but this difference was significant only after adjustment for age (OR = 0.36, 95% CI = 0.15-0.88, *P* = 0.026).

To evaluate the combined effect of both *PON1* SNPs on NT-proBNP, haplotype analysis was performed. Three haplotypes were observed in our study: *PON1* TA, AA in AG (SNP order from 5′-end to 3′-end: rs854560, rs662) and their estimated frequencies were 0.366, 0.366, and 0.267, respectively. Compared to reference *PON1* TA haplotype, carriers of *PON1* AG haplotype were significantly more likely to have increased NT-proBNP (OR = 5.48, 95% CI = 2.10-14.29, *P* < 0.001). On the other hand, *PON1* AA haplotype was not associated with NT-proBNP (OR = 1.33, 95% CI = 0.66-2.69, *P* = 0.418).

### 3.2. LVEF

Operation and RT side was not associated with LVEF reduction (right vs. left: OR = 1.15, 95% CI = 0.29-4.54, *P* = 0.846). Other clinical characteristics, including chemotherapy parameters, were also not associated with LVEF reduction in our study (all *P* > 0.05). None of the investigated SNPs was associated with LVEF (Table 4).

### 3.3. NYHA

Among clinical parameters, higher NYHA class was associated with higher body mass index (BMI) (OR = 1.20, 95% CI = 1.05-1.38, *P* = 0.006) and presence of hyperlipidemia (OR = 4.60, 95% CI = 1.39-15.19, *P* = 0.012). Operation and RT side was not associated with NYHA class (right vs. left: OR = 0.77, 95% CI = 0.27-2.19, *P* = 0.624). Other clinical characteristics, including chemotherapy parameters, were also not associated with NYHA class in our study (all *P* > 0.05).

Carriers of at least one polymorphic *CAT* rs1001179 allele were significantly more likely to be NYHA class 2 (OR = 4.09, 95% CI = 1.37-12.25, *P* = 0.012), even after adjustment for hyperlipidemia and BMI (OR = 4.14, 95% CI = 1.22-14.09, *P* = 0.023). Other SNPs were not associated with NYHA class in univariable or multivariable analysis (Table 4).

Among patients with left-sided breast cancer only, 9 (18.8%) patients were NYHA class 2. Carriers of at least one polymorphic *CAT* rs1001179 allele were still significantly more likely to be NYHA class 2 (OR = 5.09, 95% CI = 1.08-24.02, *P* = 0.040) in univariable analysis, while only a trend was observed after adjustment for hyperlipidemia and BMI (OR = 5.94, 95% CI = 0.84-42.22, *P* = 0.075). Other SNPs were also not associated with NYHA class in univariable or multivariable analysis in left-sided breast cancer (all *P* > 0.05).

## 4. Discussion

In the present study, we evaluated the association of genetic variability in antioxidative genes with cardiotoxicity in HER2-positive early breast cancer patients treated with adjuvant RT and trastuzumab. We showed that *PON1* rs854560 and rs662 as well as *GSTP1* rs1695 polymorphisms were associated with NT-proBNP levels, while *CAT* rs1001179 was associated with NYHA class.

Cardiotoxicity of breast cancer treatment has been widely investigated in recent years as improvements in cancer treatment that led to improved long-term survival also increased treatment-related cardiotoxicity [18]. Both systemic therapy and RT have been associated with increased risk of cardiac adverse events [18]. In our study, more than one third of patients exhibited signs of cardiotoxicity after treatment. Differences in systemic therapy or RT parameters among patients were not associated with any of the investigated cardiotoxicity parameters. Among other characteristics, the most important clinical predictor of increased NT-proBNP was higher age, which is consistent with other studies and is reflected also in reference ranges for healthy individuals [50]. BMI has been reported to be associated with NT-proBNP [50], but we did not observe any association with factors related to cardiovascular diseases such as BMI, smoking, hyperlipidemia or hypertension, or other clinical parameters. On the other hand, presence of hyperlipidemia and higher BMI were associated with mild symptoms of heart failure according to NYHA classification. Interestingly, none of the patients' or treatment characteristics were associated with important LVEF reduction.

Our results suggest that *PON1* genetic variability was the most important predictor of treatment-related cardiotoxicity in HER2-positive early breast cancer patients. The key observation was the association of *PON1* polymorphisms with NT-proBNP: polymorphic *PON1* rs854560 T allele was associated with lower NT-proBNP levels, while *PON1* polymorphic rs662 G allele was associated with higher NT-proBNP levels. Additionally, an even larger association with increased NT-proBNP was observed in *PON1* AG haplotype combining both normal rs854560 and polymorphic rs662 allele associated with higher NT-proBNP in single SNP analysis.

PON1 is a plasma enzyme located in HDL that has antioxidative, antiatherosclerotic, and anti-inflammatory role [51]. PON1 inhibits LDL oxidation, prevents accumulation of oxidized LDL, and stimulates cholesterol efflux from macrophages [51, 52]. PON1 activity is inversely correlated with cardiovascular diseases [45, 51, 52]. Several functional polymorphisms were identified in the *PON1* gene. *PON1* rs854560 is a nonsynonymous SNP, and the leucine to methionine substitution was previously associated with increased enzyme activity and serum concentration [45, 53, 54]. *PON1* rs662 is also a nonsynonymous SNP, leading to a glutamine to arginine substitution with the biggest impact on enzyme activity [53]. Polymorphic rs662 G allele results in lower enzymatic activity that limits PON1 capacity for lipid peroxide hydrolysis and therefore less effectively inhibits LDL oxidation [45, 54]. Interestingly, rs662 has been associated with a substrate-specific change in enzyme activity: the polymorphic allele was associated with increased paraoxonase activity, while hydrolytic activity towards other substrates was lower [45, 53, 55]. *PON1* rs662 was also associated with increased LDL and decreased HDL concentrations [56, 57]. The association of *PON1* rs854560 with lipoprotein levels is less pronounced and might also vary in different pathologies [51]. This data is in concordance with our results, as the polymorphic rs854560 T allele, associated with higher enzyme activity, was also associated with lower cardiotoxicity, while the polymorphic rs662 G allele, associated with lower enzyme activity, was associated with increased cardiotoxicity in our study.

A lot of studies also investigated the influence of *PON1* polymorphisms on the risk of developing different cardiovascular diseases. Generally, *PON1* rs662 was associated with slightly increased cardiovascular disease risk, especially in the recessive genetic model, while *PON1* rs854560 was associated with somewhat decreased risk in meta-analyses [58–62], consistent with our results. However, some studies found no significant associations and differences were observed among different populations [58, 59]. On the other hand, several studies also investigated the role of *PON1* polymorphisms in cancer risk. Despite discrepancies among different studies, latest meta-analyses suggest rs662 is associated with lower breast cancer risk, while rs854560 is associated with increased breast cancer risk [63–66]. These results also suggest rs662 and rs854560 have an opposite effect, but further studies are needed as different results were observed in other cancer types [65].

Studies suggest serum PON1 level and its activity are lower in cancer patients, including breast cancer [67–70], but only a few studies investigated the role of PON1 in cancer treatment response or toxicity. So far, no studies investigated the association of PON1 with response to trastuzumab or anthracyclines, while a few studies focusing on RT were already published [55, 69, 70]. In breast cancer patients treated with adjuvant RT, PON1 concentration and activity increased after RT with significant differences observed among different molecular subtypes [70]. In luminal B (HER2-positive) subtype, PON1 concentration after RT was lower compared to luminal A subtype. HER2 expression was also associated with altered expression of other antioxidative enzymes, which could modify the risk for cardiotoxicity [70, 71]. In lung cancer, as well as head and neck cancer patients treated with RT, PON1 concentration also increased after RT [69]. Additionally, in patients with nasopharyngeal carcinoma, the combination of *PON1* rs662 and another polymorphism, rs705379 (c.-108C>T) was associated with carotid atherosclerosis after RT of the neck, while *PON1* rs854560 was not investigated [55]. In contrast to other studies, rs662 was associated with lower carotid plaque scores, which could be partly due to differences in activities observed for different substrates [55]. Better evaluation of *PON1* genetic variability, concentration or activity is therefore needed to improve the understanding of PON1 role in cardiovascular disease and especially in treatment-related cardiotoxicity.

In our study, polymorphic *GSTP1* rs1695 A allele was also significantly associated with lower risk for increased NT-proBNP after accounting for age. GSTP1 is involved in detoxification of xenobiotics through conjugation with glutathione [72]. *GSTP1* rs1695 is a nonsynonymous SNP that leads to lower enzyme activity [73]. Several studies investigated the role of *GSTP1* rs1695 in breast cancer susceptibility or response to treatment [73–79]. Latest meta-analyses suggest this SNP might contribute to increased breast cancer risk; however, the association was significant only in specific populations [73, 76, 77]. *GSTP1* polymorphisms with lower enzyme activity were also proposed to be associated with better treatment outcome [78, 79], and based on meta-analysis results, *GSTP1* rs1695 could serve as a predictor of response to anthracycline-based chemotherapy [78]. Additionally, this could also lead to altered risk of treatment-related toxicity. In a previous study, *GSTP1* rs1695 was not associated with LVEF reduction after treatment with anthracyclines, consistent with our results, while NT-proBNP was not evaluated [80]. No studies investigated the association of *GSTP1* with cardiotoxicity of treatment with RT or trastuzumab. However, rs1695 was previously associated with increased heart failure and coronary artery disease risk [81, 82]. Apart from its role in response to oxidative stress and lipid peroxidation, GSTP1 may also affect different signalling pathways, which could contribute to the observed association with heart disease [81]. Additional studies are needed to better understand the role of GSPT1 and its potential interaction with other clinical parameters in cardiotoxicity of breast cancer treatment, especially in patients treated with RT.

Carriers of at least one polymorphic *CAT* rs1001179 A allele were significantly more likely to exhibit mild symptoms of heart failure according to NYHA classification in our study, even after taking into account hyperlipidemia and BMI. This association was also observed in univariable analysis in the subgroup of left-sided breast cancer patients. In these cases, the heart lies directly below the target tissue for irradiation. *CAT* rs1001179 is located in the promoter region of the gene and was previously associated with lower expression and activity of catalase and thus could confer worse defence against oxidative stress [43, 83]. Overall, the role of *CAT* genetic variability in breast cancer is not well known, with previous studies suggesting rs1001179 is not associated with breast cancer risk [84]. Only a handful of studies have previously investigated the role of *CAT* genetic variability in cardiotoxicity of anthracyclines, while so far, no study has investigated cardiotoxicity of treatment with RT or trastuzumab [85–87]. In one study, intronic SNP *CAT* rs10836235 was associated with cardiac damage in childhood acute leukemia patients treated with anthracyclines, but no significant association with *CAT* rs1001179 was observed [85]. GWAS and meta-analyses did not identify catalase as a risk factor for cardiotoxicity of anthracyclines [86, 87]. Studies investigating the role of *CAT* polymorphisms in cardiovascular disease are also scarce; however, *CAT* rs1001179 was not a risk factor for coronary artery disease in a recent study [88]. Further studies are therefore needed to elucidate the role of catalase in the development of cardiotoxicity of breast cancer treatment after different treatment modalities.

The main limitation of our study was its small sample size. However, we had clear inclusion and exclusion criteria and thus included a clinically well-defined study group of patients with a HER2-positive breast cancer subtype with thorough evaluation of cardiotoxicity parameters. According to the available literature, we were the first to evaluate the role of genetic variability in cardiotoxicity after adjuvant RT in HER2-positive breast cancer patients. Another limitation of our study is the fact that we had to exclude *SOD2* rs4880 from the analysis as it was not in agreement with HWE. As *SOD2* rs4880 was marginally associated with breast cancer risk in Caucasians, this could contribute to the observed deviation from HWE [89]. Still, we were among the first to assess the influence of genetic variability of several antioxidative genes on cardiotoxicity of breast cancer treatment and the first to show that especially *PON1* polymorphisms could contribute to the occurrence of cardiac adverse events. As our patients were treated with different treatment modalities that all contribute to cardiotoxicity, studies on patients treated only with RT or with only one type of systemic therapy could help elucidate the role of the investigated polymorphisms. Larger studies are therefore needed to further validate our results.

## 5. Conclusions

In conclusion, our study indicates that functional polymorphisms in antioxidative genes might serve as biomarkers of treatment-related cardiotoxicity in breast cancer patients. Better understanding of adverse events could improve patient management and affect the health and quality of life of breast cancer patients. In the future, genetic markers could contribute to the personalization of RT and systemic therapy in breast cancer patients.

## Figures and Tables

**Table 1:** Characteristics of breast cancer patients included in the study (*N* = 101) and treatment parameters.

Characteristic	Category/Unit	*N* (%)
Age	Years	50.9 (42.1-59.1)^∗^
Type of surgery	Mastectomy	48 (47.5)
	Conservative surgery	53 (52.5)
Side of surgery	Left	48 (47.5)
	Right	53 (52.5)
Tumour type	Invasive lobular carcinoma	2 (2.0)
	Invasive ductal carcinoma	96 (95.0)
	Other	3 (3.0)
Cancer grade	1	1 (1.0)
	2	31 (30.7)
	3	69 (68.3)
Chemotherapy scheme	AC/EC/FAC/FEC with taxanes	54 (53.5)
	AC/EC/FAC/FEC without taxanes	43 (42.6)
	Other	4 (4.0)
Anthracyclines	Yes	99 (98.0)
	Doxorubicin	6 (6.0)
	Epirubicin	93 (92.1)
	No	2 (2.0)
Anthracyclines cumulative dose	Doxorubicin, mg/m^2^ BSA	342 (318-413)^∗^
	Epirubicin, mg/m^2^ BSA	353 (294-522)^∗^
Taxanes	Yes	58 (57.4)
	Paclitaxel	17 (16.7)
	Docetaxel	41 (40.6)
	No	43 (42.6)
Taxanes cumulative dose	Paclitaxel, mg/m^2^ BSA	886 (739-938)^∗^
	Docetaxel, mg/m^2^ BSA	286 (269-299)∗
Hormonal therapy	Yes	57 (56.4)
	No	44 (43.6)
Treatment scheme of RT	25 × 2 Gy	84 (83.2)
	17 or 18 × 2.5 Gy	17 (16.8)
Site of RT	Breast/mammary region	58 (57.4)
	(Breast/mammary region) + regional lymph nodes	43 (42.6)
RT technique	2D RT	80 (79.2)
	3D CRT	14 (13.9)
	Electrons	7 (6.9)
Hypertension	Yes	29 (28.7)
	No	72 (71.3)
Hyperlipidemia	Yes	21 (20.8)
	No	80 (79.2)
Smoking	Yes	16 (15.8)
	No	85 (84.2)
Diabetes	Yes	1 (1.0)
	No	100 (99.0)
Body mass index	kg/m^2^	27.1 (24.3-29.7)∗

^∗^median (25%-75%). 2D RT: two-dimensional radiotherapy; 3D CRT: three-dimensional conformal radiotherapy; AC: doxorubicin, cyclophosphamide; BSA: body surface area calculated according to the Du Bois formula; EC: epirubicin, cyclophosphamide; FAC: 5-fluorouracil, doxorubicin, cyclophosphamide; FEC: 5-fluorouracil, epirubicin, and cyclophosphamide; Gy: Gray; RT: radiotherapy.

**Table 2:** Markers of cardiac side effects of breast cancer therapy.

Marker	Category/Unit	*N* (%)
Initial LVEF	%	65 (60-70)^∗^
Absolute change in LVEF	%	3 (-3 do 9)^∗^
LVEF reduction	No	92 (91.1)
	Yes	9 (8.9)
NT-proBNP	ng/l	90 (56-157)^∗^
NT-proBNP	<125 ng/l	65 (64.4)
	≥125 ng/l	36 (35.6)
NYHA	Class 1	84 (83.2)
	Class 2	17 (16.8)

^∗^median (25%-75%). LVEF: left ventricular ejection fraction; NT-proBNP: N-terminal pro B-type natriuretic peptide; NYHA: New York Heart Association. Absolute change in LVEF was calculated as the difference between LVEF after completed adjuvant radiotherapy and trastuzumab treatment, and LVEF before radiotherapy. LVEF reduction was classified as a decrease of LVEF for ≥10% or a final value of LVEF <50%.

**Table 3:** Association of polymorphisms in antioxidative genes with NT-proBNP levels.

SNP	Genotype	NT-proBNP median (25-75%)	*P*	NT-proBNP <125 ng/l, *N* (%)	NT-proBNP ≥125 ng/l, *N* (%)	OR (95% CI)	*P*	OR (95% CI)_adj_	*P*_adj_
*PON1* rs854560	AA	126 (59-200)		21 (50.0)	21 (50.0)	Reference		Reference	
	AT+TT	79 (51-125)	**0.048**	44 (74.6)	15 (25.4)	0.34 (0.15-0.79)	**0.012**	0.35 (0.15-0.83)	**0.017**
*PON1* rs662	AA	79 (47.75-118.5)		43 (79.6)	11 (20.4)	Reference		Reference	
	AG+GG	135 (60-193)	**0.007**	22 (46.8)	25 (53.2)	4.44 (1.85-10.66)	**0.001**	5.41 (2.12-13.78)	**<0.001**
*GSTP1* rs1138272	CC	92 (58-156)		53 (63.9)	30 (36.1)	Reference		Reference	
	CT+TT	78.5 (36-165.5)	0.407	12 (66.7)	6 (33.3)	0.88 (0.30-2.56)	0.821	0.71 (0.23-2.16)	0.545
*GSTP1* rs1695	GG	122 (65.75-174.5)		24 (54.5)	20 (45.5)	Reference		Reference	
	GA+AA	77 (48.5-144)	0.101	41 (71.9)	16 (28.1)	0.47 (0.21-1.07)	0.073	0.36 (0.15-0.88)	**0.026**
*CAT* rs1001179	GG	88 (52.5-148.25)		42 (65.6)	22 (34.4)	Reference		Reference	
	GA+AA	96 (56-182)	0.680	23 (62.2)	14 (37.8)	1.16 (0.50-2.70)	0.762	0.99 (0.41-2.36)	0.973
*HIF1A* rs1154965	CC	91 (56-154.5)		56 (63.6)	32 (36.4)	Reference		Reference	
	CT+TT	79 (54.5-180)	0.666	9 (69.2)	4 (30.8)	0.78 (0.22-2.73)	0.695	0.87 (0.24-3.13)	0.827

Adj: adjusted for age; CI: confidence interval; NT-proBNP: N-terminal pro B-type natriuretic peptide; OR: odds ratio.

**Table 4:** Association of polymorphisms in antioxidative genes with NYHA class and LVEF reduction.

		NYHA						LVEF reduction			
SNP	Genotype	Class 1, *N* (%)	Class 2, *N* (%)	OR (95% CI)	*P*	OR (95% CI)_adj_	*P*_adj_	No, *N* (%)	Yes, *N* (%)	OR (95% CI)	*P*
*PON1* rs854560	AA	33 (78.6)	9 (21.4)	Reference				38 (90.5)	4 (9.5)	Reference	
	AT+TT	51 (86.4)	8 (13.6)	0.58 (0.20-1.64)	0.301	0.61 (0.19-1.99)	0.416	54 (91.5)	5 (8.5)	0.88 (0.22-3.49)	0.885
*PON1* rs662	AA	44 (81.5)	10 (18.5)	Reference				50 (92.6)	4 (7.4)	Reference	
	AG+GG	40 (85.1)	7 (14.9)	0.77 (0.27-2.22)	0.628	0.88 (0.27-2.84)	0.832	42 (89.4)	5 (10.6)	1.49 (0.38-5.90)	0.572
*GSTP1* rs1138272	CC	69 (83.1)	14 (16.9)	Reference				75 (90.4)	8 (9.6)	Reference	
	CT+TT	15 (83.3)	3 (16.7)	0.99 (0.25-3.87)	0.984	0.88 (0.20-3.92)	0.862	17 (94.4)	1 (5.6)	0.55 (0.07-4.71)	0.586
*GSTP1* rs1695	GG	36 (81.8)	8 (18.2)	Reference				38 (86.4)	6 (13.6)	Reference	
	GA+AA	48 (84.2)	9 (15.8)	0.84 (0.30-2.40)	0.750	0.84 (0.26-2.74)	0.773	54 (94.7)	3 (5.3)	0.35 (0.08-1.50)	0.157
*CAT* rs1001179	GG	58 (90.6)	6 (9.4)	Reference				58 (90.6)	6 (9.4)	Reference	
	GA+AA	26 (70.3)	11 (29.7)	4.09 (1.37-12.25)	**0.012**	4.14 (1.22-14.09)	**0.023**	34 (91.9)	3 (8.1)	0.85 (0.20-3.63)	0.830
*HIF1A* rs1154965	CC	71 (80.7)	17 (19.3)	Reference				81 (92.0)	7 (8.0)	Reference	
	CT+TT	13 (100.0)	0 (0.0)	/	0.118^∗^			11 (84.6)	2 (15.4)	2.10 (0.39-11.44)	0.389

Adj: adjusted for hyperlipidemia and body mass index. ^∗^calculated using Fisher's exact test. CI: confidence interval; LVEF: left ventricular ejection fraction; NYHA: New York Heart Association; OR: odds ratio.

## Data Availability

The data used to support the findings of this study are included within the article and supplementary information file.

## Supplementary Materials

Supplementary Materials Supplementary Table 1: genotype frequencies of investigated polymorphisms.
