# Supplementary material for: Genetic Variability of Antioxidative Mechanisms and Cardiotoxicity after Adjuvant Radiotherapy in HER2-Positive Breast Cancer Patients
Source: Dis Markers. 2020 Dec 19;2020:6645588. doi: 10.1155/2020/6645588 (PMC7772014; doi:10.1155/2020/6645588)
Supplement: Supplementary Materials — Supplementary Table 1: genotype frequencies of investigated polymorphisms. [file 6645588.f1.pdf]

Supplementary Table 1: Genotype frequencies of investigated polymorphisms.

Gene	Polymorphism	Role	Genotype	N (%)	MAF	pHWE
PON1	rs854560	p.Leu55Met	AA	42 (41.6)	0.366	0.536
			AT	44 (43.6)		
			TT	15 (14.9)		
PON1	rs662	p.Gln192Arg	AA	54 (53.5)	0.267	0.912
			AG	40 (39.6)		
			GG	7 (6.9)		
GSTP1	rs1138272	p.Ala114Val	CC	83 (82.2)	0.099	0.260
			CT	16 (15.8)		
			TT	2 (2.0)		
GSTP1	rs1695	p.Ile105Val	AA	44 (43.6)	0.317	0.149
			AG	50 (49.5)		
			GG	7 (6.9)		
SOD2	rs4880	p.Ala16Val	CC	35 (34.7)	0.475	0.004
			TC	36 (35.6)		
			TT	30 (29.7)		
CAT	rs1001179	c.-330C>T	CC	64 (63.4)	0.203	0.921
			CT	33 (32.7)		
			TT	4 (4.0)		
HIF1A	rs1154965	p.Pro582Ser	CC	88 (87.1)	0.069	0.427
			CT	12 (11.9)		
			TT	1 (1.0)		

HWE, Hardy-Weinberg equilibrium; MAF, minor allele frequency
